# Comparison of push‐out bond strength of endodontic sealers after root canal drying with different techniques

**DOI:** 10.1002/cre2.720

**Published:** 2023-02-19

**Authors:** Ahmadreza Sarrafan, Ali Soleymani, Tasnim Bagheri Chenari, Seyedali Seyedmajidi

**Affiliations:** ^1^ Department of Endodontics, School of Dentistry Babol University of Medical Sciences Babol Iran; ^2^ Department of Endodontics, School of Dentistry, Dental Materials Research Center, Health Research Institute Babol University of Medical Sciences Babol Iran; ^3^ Department of Endodontics, School of Dentistry, Oral Health Center, Health Research Institute Babol University of Medical Sciences Babol Iran; ^4^ School of Dentistry, Dental Materials Research Center, Health Research Institute Babol University of Medical Sciences Babol Iran

**Keywords:** epoxy resin‐based root canal sealer, ethanol, mineral trioxide aggregate, tooth root

## Abstract

**Objectives:**

An ideal endodontic sealer should bond to both dentin and root‐filling material. This study aimed to assess the push‐out bond strength (PBS) of mineral trioxide aggregate (MTA)‐Fillapex, Endoseal MTA, AH26, and Sure‐Seal Root to root dentin after root canal drying with different techniques.

**Materials and Methods:**

This in vitro study was conducted on 160 extracted mandibular premolars. After root canal preparation, the teeth were randomly divided into four groups (*n* = 40) of drying with ethanol, paper point, air vacuum, and control (wet canal). Each group was divided into four subgroups (*n* = 10) for use of AH26, Sure‐Seal Root, MTA‐Fillapex, and Endoseal MTA sealers. The mean PBS was measured by a universal testing machine. The mode of failure was determined under a stereomicroscope. Data were analyzed by the Kruskal–Wallis and Games–Howell tests.

**Results:**

The maximum mean PBS was equally recorded in ethanol and paper point groups, and was significantly higher than that of control and air vacuum groups (*p* < .001). In the use of AH26 sealer, the mean PBS in drying with ethanol was significantly higher than all other methods (*p* < .05). The mean PBS in drying with a paper point was also significantly higher compared with control and air vacuum methods (*p* < .05). In the use of Sure‐Seal, the mean PBS in drying with a paper point was significantly higher than other methods (*p* < .05). The mean PBS in drying with ethanol was significantly higher than that in control and air vacuum methods (*p* < .001). In the use of MTA‐Fillapex and Endoseal‐MTA, the technique of drying had no significant effect on PBS. Adhesive and mixed failures were the most common in all drying groups.

**Conclusions:**

Drying with ethanol and paper point enhanced the PBS of sealers to root dentin.

## 1 INTRODUCTION

A successful endodontic treatment highly depends on the durability and biocompatibility of root‐filling materials, that is, gutta‐percha and sealer. Endodontic sealers are applied to seal the gap between the root dentin and root filling material and fill the root canal wall irregularities, accessory canals, and voids between gutta‐percha points in the lateral compaction obturation technique. Also, sealers serve as a lubricant in the process of root canal obturation (Saleh et al., 2003).

The push‐out bond strength (PBS) of sealers to root dentinal walls is an influential factor in determining the success rate of endodontic sealers (Saleh et al., 2003; C. S. Teixeira et al., 2009; F. B. Teixeira et al., 2004). Optimal PBS of sealer to root dentin enhances the tensile strength, prevents the microleakage of materials and microorganisms, further stabilizes the root filling material, and improves the clinical service (C. S. Teixeira et al., 2009). Different sealers have variable adhesive properties in bonding to dentin. A number of factors are responsible for different adhesive properties of sealers, such as the differences in dentin structure of different teeth or even different parts of the same tooth, presence/absence of smear layer, the chemical composition of sealer, and dentin reaction (Eldeniz et al., 2005; Lee et al., 2002; Tagger et al., 2002).

The level of dentin moisture before sealer application is another influential factor on the bond strength of sealers to dentin (Nagas et al., 2012). Evidence shows that the residual moisture in the canal adversely affects the adhesion of resin sealers (Rijal, 2016). However, it does not mean that the root canal walls should be completely dried out. Root dentin should remain slightly moist so that the sealer can use its hydrophilic property for attachment to root dentin (Jang et al., 2010). Thus, the effect of excess moisture on the tensile bond strength of sealer to root dentin should be investigated.

Drying the root canal with paper points is the most commonly practiced technique for root canal drying. Paper points highly absorb moisture, and are flexible while having adequate stiffness. Thus, drying the root canals with paper points is the most popular and simplest technique for root canal drying. According to the Marangoni conversion of the power law, ethanol increases the evaporation power of water, and evidence shows that the use of ethanol decreases the residual moisture and expedites the root canal drying process (Jang et al., 2010; Rijal, 2016). Nonetheless, mechanical devices such as EndoVac® (Paula et al., 2016) and air vacuum (Luer Vacuum adapter) (Nagas et al., 2012) have also been proposed for root canal drying.

Several types of endodontic sealers with different chemical compositions are currently available in the dental market. Mineral trioxide aggregate (MTA)‐based sealers were introduced to the market almost recently. The presence of MTA in the composition of such sealers is their major strength, which is responsible for their optimal biocompatibility. Endoseal MTA (Endoseal; Maruchi) and MTA‐Fillapex (Angelus) are among such sealers. Their chemical composition includes resin, bismuth oxide, silicate nanoparticles, and gypsum. Radiopacity, low solubility, and polymerization expansion are among the favorable properties of MTA‐based sealers (Assmann et al., 2012; Camilleri, 2009).

Sure‐Seal Root (Gyeonggi‐do, South Korea) is a novel calcium silicate‐based sealer, which can bond to hydroxyapatite and is hydrophilic (Huang et al., 2017).

AH26 sealer is a bis‐phenol epoxy resin sealer. Low shrinkage is an advantage of AH26. However, it releases formaldehyde following polymerization, which is a major drawback of this sealer (Ashraf et al., 2022).

Considering the significant role of moisture in the successful bonding of sealers to root dentin, and the lack of previous studies on the effect of moisture on PBS of the aforementioned sealers, this study aimed to assess the PBS of MTA‐Fillapex, Endoseal MTA, AH26, and Sure‐Seal Root to root dentin after root canal drying with different techniques. The null hypotheses of the study were as follows: (I) the root canal drying technique would have no significant effect on PBS of sealers to root dentin; (II) the PBS of different sealers used with the same drying technique would not be significantly different.

## 2 MATERIALS AND METHODS

This in vitro, experimental study was conducted on 160 single‐rooted mandibular premolars extracted due to periodontal reasons or impaction. The study protocol was approved by the ethics committee of Babol University of Medical Sciences (IR.MUBABOL.HRI.REC.1400.016).

### 2.1 Sample size

The minimum sample size was calculated to be eight teeth in each group according to a study by Kapur et al. (2019) and assuming 95% accuracy (*α* = 0.05), and study power of 80% using the following formula. To increase the accuracy, 10 teeth were assigned to each group.

$$n>\frac{{\left(Z_{1-\alpha /2}+Z_{1-\beta }\right)}^{2}(S_{1}^{2}+S_{2}^{2})}{{\left({µ}_{1}-{µ}_{2}\right)}^{2}}.$$

### 2.2 Eligibility criteria

The inclusion criteria were sound caries‐free teeth with no root curvature, no root fracture, closed apices, absence of internal/external root resorption, and no history of previous endodontic treatment (Dias et al., 2014). Teeth with developmental defects in their roots were excluded. The teeth underwent periapical radiography to ensure the eligibility criteria. All teeth were then stored in saline for 1 month.

### 2.3 Specimen preparation

At the time of the experiment, the anatomical crown was cut at the cementoenamel junction by a fissure carbide bur, and the root canals were instrumented starting with a #10 K‐file until #20 by the step‐back technique. Next, the root canals were prepared with ProTaper Universal rotary system (Dentsply, Maillefer) to F3 (Sadat Shojaee et al., 2019). The root canals were irrigated with 10 mL of 5.25% sodium hypochlorite after using each file. Finally, the smear layer was removed by using 17% ethylenediaminetetraacetic acid for 1 min followed by a final rinse with 10 mL of distilled water to eliminate the chemical agents from the root canal system. Apical patency was ensured visually by injecting distilled water into the canal through the coronal access and observing its apical extrusion (Nagas et al., 2012). After the final rinse, the roots were randomly divided into four groups (*n* = 40) according to the study by Zmener et al. (2008).

Group 1. Drying with ethanol: Excess moisture was removed from the root canal system by using #30 paper points (Biomed Meta). Next, 10 mL of 95% ethanol was injected into the canal to the working length using a tuberculin syringe with a 30‐gauge needle. Ethanol was gradually injected into the root canal while the syringe was gradually pulled back. Next, the ethanol seal with distilled water was visually confirmed. It remained in the canal for 10 s, and then excess ethanol was removed by a paper point until no moisture was visible.

Group 2. Paper point (natural moisture): The root canals were dried with paper points until no moisture was observed on the surface.

Group 3. Luer Vacuum (moist): Luer Vacuum (Ultradent) was used with gentle pecking motion in the canal for 5 s, and then a paper point was used for 1 s.

Group 4. Control group (wet): The root canals were not dried in this group (Dias et al., 2014).

Next, the root canals in all groups were obturated with gutta‐percha (F3; Meta BioMed) and sealer. Depending on the type of sealer, each group was randomly divided into four subgroups (*n* = 10): AH‐26 (Dentsply DeTrey) was used in subgroup 1 (supplied as powder/liquid), MTA‐Fillapex (Angelus) was used in subgroup 2 (supplied as paste/paste), Endoseal MTA (Maruchi) was used in subgroup 3 (automix syringe), and Sure‐Seal Root (Sure Dent Corp) was used in subgroup 4 (automix syringe). Thus, a total of 16 subgroups (*n* = 10) were evaluated. Obturation was performed with a single cone obturation technique in all groups.

### 2.4 Evaluation of PBS

After completion of obturation, the roots were incubated at 37°C and 100% humidity for 24 h (F. B. Teixeira et al., 2004). After incubation, two transverse sections were made with 1.5 mm diameter and 1 mm height (thickness) at the middle third of the roots by a low‐speed diamond disc under water coolant using a digital cutting machine (Nemo). For the purpose of standardization, the diameter of each disc was measured under a stereomicroscope (Dewinter). To measure the PBS, each disc was mounted on a jig such that the coronal surface of the disc faced the metal surface of the jig. A cylindrical plugger with a 1 mm diameter was positioned at the center of the root canal space in each disc to prevent the contact of metal with dentin surrounding the root filling material. The load was applied by the tip of the cylindrical plugger to the disc in apicocoronal direction in a universal testing machine (Koopa) with a 200‐kg load cell at a crosshead speed of 1 mm/minute. The minimum force required for dislodgement of filling material and its debonding from the root canal wall was recorded as PBS. The value was quantified using the following formula: *A* = 2Πr × h where r indicates the root canal radius measured under a stereomicroscope, and h is the thickness of the specimen in millimeters. Finally, the PBS was calculated using the following formula: *P* = *F*/*A*.

### 2.5 Analysis of failure modes

**Figure 1:**
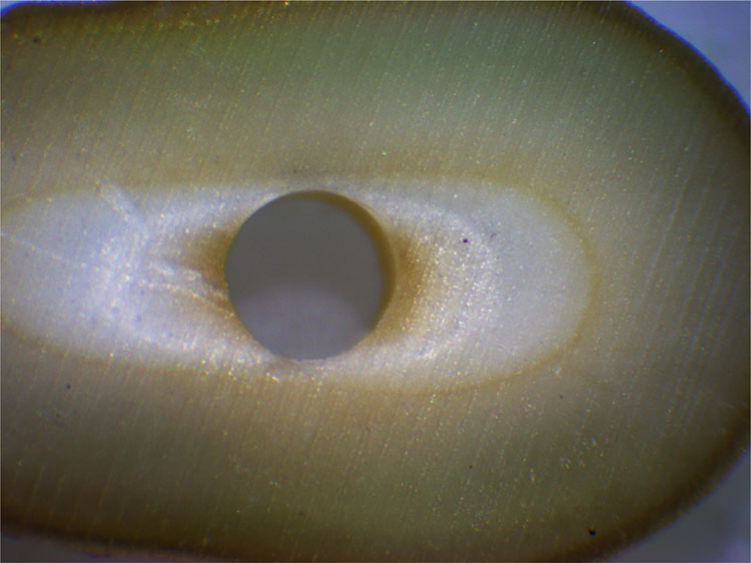
Adhesive failure in mineral trioxide aggregate Fillapex with no drying.

The mode of failure was evaluated under a stereomicroscope (Dewinter) at ×10 magnification, and classified as follows:

Adhesive failure: Debonding at the bonding interface (Figure 1).

**Figure 2:**
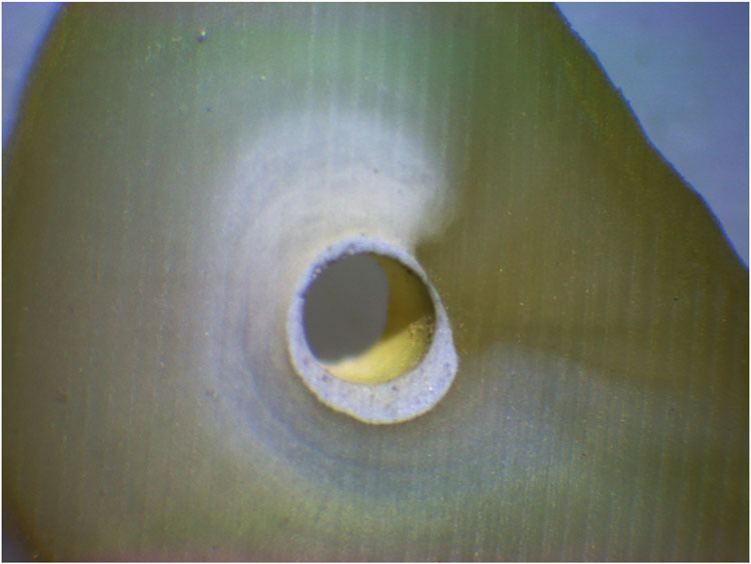
Cohesive failure in SureSeal Root in drying with ethanol.

Cohesive failure: Fracture within the dentin structure or root filling material (Figure 2).

**Figure 3:**
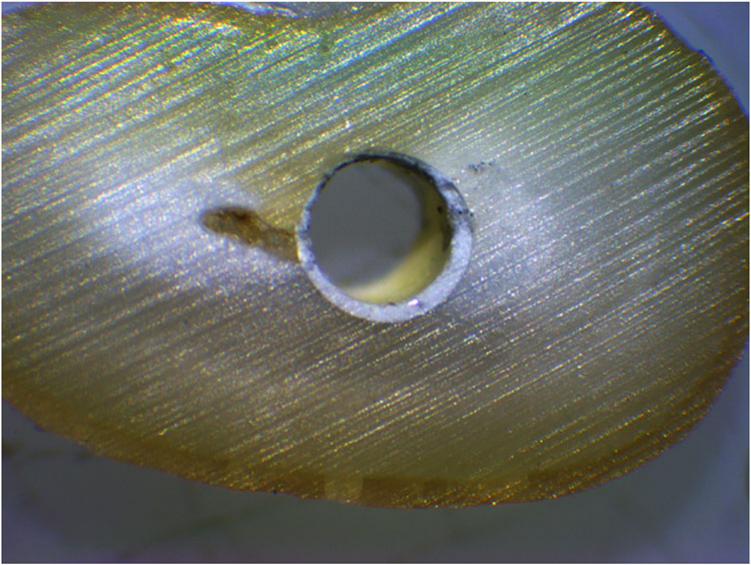
Mixed failure in AH 26 in drying with air vacuum.

Mixed failure: A combination of adhesive and cohesive failures (Tagger et al., 2002) (Figure 3).

The frequency of different modes of failure was reported for each group in percentage.

### 2.6 Statistical analysis

Data were analyzed by SPSS version 22. The Kolmogorov–Smirnov test was used to assess the normality of data distribution, which showed a non‐normal distribution of data (*p* < .05). Thus, the groups were compared with the Kruskal–Wallis test followed by the Games–Howell test for pairwise comparisons at *p* < .05 level of significance.

## 3 RESULTS

Table 1 presents the mean PBS in different drying techniques based on the type of sealer used.

**Table 1:** Mean PBS in different drying techniques based on the type of sealer.

Sealers	Control (*n* = 10) mean ± std. deviation	Paper point (*n* = 10) mean ± std. deviation	Ethanol (*n* = 10) mean ± std. deviation	Air vacuum (*n* = 10) mean ± std. deviation	*p* Value^a^
AH26 (*n* = 40)	1.31 ± 0.61	3.10 ± 1.88	5.61 ± 2.27	1.41 ± 0.67	<.001
MTA‐Fillapex (*n* = 40)	1.70 ± 1.00	1.82 ± 1.32	1.46 ± 1.12	1.07 ± 0.56	.175
Endoseal MTA (*n* = 40)	2.36 ± 2.21	1.94 ± 1.39	2.73 ± 1.78	1.52 ± 0.79	.077
SureSeal (*n* = 40)	1.72 ± 1.28	8.07 ± 3.14	4.69 ± 1.76	1.34 ± 0.82	<.001
*p* Value^a^	.841	<.001	<.001	.04	‐

Abbreviations: MTA, mineral trioxide aggregate; PBS, push‐out bond strength.

^a^ Kruskal–Wallis test.

### 3.1 Comparison of PBS of different drying techniques irrespective of sealer type

The maximum mean PBS was noted in drying with ethanol followed by a paper point, control, and air vacuum groups. The maximum mean PBS was equally recorded in ethanol and paper point groups, followed by control, and air vacuum groups. The difference in the mean PBS was significant among the four groups (*p* < .001). Pairwise comparisons showed that the mean PBS in drying with ethanol and paper point techniques was not significantly different (*p* > .05). However, the mean PBS of ethanol and paper point groups was significantly higher than that of control and air vacuum groups (*p* < .001). Thus, ethanol and paper points equally ranked first regarding the best drying technique.

### 3.2 Comparison of PBS of different drying techniques based on the sealer type

AH26: In the use of AH26 sealer, the highest mean PBS was noted in drying with ethanol, followed by paper point, control, and air vacuum techniques. According to the Kruskal–Wallis test, the difference in the mean PBS of different drying techniques was significant in the use of AH26 sealer (*p* < .001). The mean PBS in drying with ethanol was significantly higher than that in the control (*p* < .001), paper point (*p* = .003), and air vacuum (*p* < .001) techniques. The mean PBS in drying with a paper point was significantly higher than that in the control (*p* = .003) and air vacuum (*p* = .005) techniques. The difference in PBS between the control and air vacuum techniques was not significant (*p* > .05).

MTA‐Fillapex: In the use of MTA‐Fillapex, the technique of drying had no significant effect on PBS (*p* = .175).

Endoseal MTA: In the use of Endoseal MTA, the technique of drying had no significant effect on PBS (*p* = .077).

SureSeal Root: In the use of SureSeal Root, the maximum PBS was noted in drying with a paper point followed by ethanol, control, and air vacuum techniques. According to the Kruskal–Wallis test, the difference in PBS of different drying techniques was significant in the use of SureSeal Root (*p* < .001). The mean PBS in drying with a paper point was significantly higher than that in the control (*p* < .001), ethanol (*p* = .001), and air‐vacuum (*p* < .001) techniques. The mean PBS in the ethanol group was significantly higher than that in the control and air vacuum groups (*p* < .001). The difference in the mean PBS of control and air vacuum groups was not significant (*p* > .05).

### 3.3 Comparison of PBS of different sealers irrespective of drying technique

The maximum mean PBS was noted in SureSeal Root followed by AH26, Endoseal MTA, and MTA‐Fillapex. The difference in this respect was significant among the four sealers as shown by the Kruskal–Wallis test (*p* < .001). Pairwise comparisons indicated that the mean PBS of SureSeal Root was significantly higher than that of MTA‐Fillapex and Endoseal MTA (*p* < .001), but had no significant difference with AH26 (*p* > .05). The mean PBS of AH26 was significantly higher than that of MTA‐Fillapex (*p* < .001), but had no significant difference with Endoseal MTA (*p* > .05). The mean PBS of Endoseal MTA was significantly higher than that of MTA‐Fillapex (*p* = .027).

### 3.4 Comparison of PBS of different sealers in the use of each drying technique

Control group: The Kruskal–Wallis test found no significant difference in PBS of different sealers in this group (*p* > .05).

Paper point group: The maximum PBS was noted in the use of SureSeal Root followed by Endoseal MTA, AH26, and MTA‐Fillapex. The difference in PBS was significant among the four sealers (Kruskal–Wallis, *p* < .001). The mean PBS of SureSeal Root was significantly higher than other sealers (*p* < .001). No other significant differences were noted (*p* > .05).

Drying with ethanol group: The maximum PBS was noted in the use of SureSeal Root followed by Endoseal MTA, AH26, and MTA‐Fillapex. The difference in PBS was significant among the four sealers (Kruskal–Wallis, *p* < .001). The mean PBS in the use of AH26 was significantly higher than that in the use of Endoseal MTA and MTA‐Fillapex (*p* < .001) but had no significant difference with SureSeal Root (*p* > .05). The mean PBS of SureSeal Root was significantly higher than that of Endoseal MTA (*p* = .006) and MTA‐Fillapex (*p* < .001). However, the mean PBS of Endoseal MTA and MTA‐Fillapex was not significantly different (*p* > .05).

Air vacuum group: The maximum PBS was noted in Endoseal MTA followed by AH26, SureSeal Root, and MTA‐Fillapex. The difference in PBS was significant among the four sealers (Kruskal–Wallis, *p* = .040). Pairwise comparisons showed that only the difference between MTA‐Fillapex and Endoseal MTA was significant (*p* = .005).

### 3.5 Mode of failure

Table 2 presents the modes of failure in the study groups. In general, in all drying techniques, the adhesive type was the most common mode of failure. However, based on the type of sealer, cohesive failure was dominant in the superior sealer, which was SureSeal Root. In drying the canal and using injectable calcium silicate sealers, higher bond strength was achieved and the mode of failure was cohesive and mixed.

**Table 2:** Frequency of different modes of failure in the groups.

Group	Mode of failure	Sealers				Total
		AH26	Fillapex	Endoseal MTA	SureSeal	
Control	Adhesive	9	13	10	5	37
	Cohesive	4	0	2	0	6
	Mixed	7	7	8	15	37
Paper point	Adhesive	15	14	7	1	37
	Cohesive	2	0	6	11	19
	Mixed	3	6	7	8	24
Ethanol	Adhesive	10	16	2	3	31
	Cohesive	2	0	10	9	21
	Mixed	8	4	8	8	28
Air Vacuum	Adhesive	12	18	4	1	35
	Cohesive	1	0	4	15	20
	Mixed	7	2	12	4	25

## 4 DISCUSSION

This study assessed the PBS of MTA‐Fillapex, Endoseal MTA, AH26, and Sure‐Seal Root to root dentin after root canal drying with different techniques. The selection of the PBS test for the present study was because it provides the most valid and accurate results. It can well simulate clinical conditions and is capable of measuring the tensile bond strength even in very low amounts (Eldeniz et al., 2005; C. S. Teixeira et al., 2009). The present results showed that the highest mean PBS was equally recorded in ethanol and paper point groups, followed by control, and air vacuum groups. The mean PBS of ethanol and paper point groups was significantly higher than that of control and air vacuum groups. Thus, the first null hypothesis of the study was rejected. This finding indicates that drying of the root canal has a fundamental role in the enhancement of PBS. Singh et al. (2019) compared the PBS in different drying techniques and reported that 70% isopropyl alcohol yielded the highest PBS. Kapur et al. (2019) discussed that the removal of excess moisture has a fundamental role in the enhancement of PBS. Paula et al. (2016) found that the use of 70% isopropyl alcohol for drying of root canal significantly increased the PBS of sealers to root dentin, compared with ethanol and EndoVac. Dias et al. (2014) reported that the application of 70% isopropyl alcohol for drying of the root canal significantly increased the PBS of sealers to root dentin. Also, Ozlek et al. (2020) demonstrated that drying of root canals, compared with no drying, significantly increased the PBS.

Several manufacturers recommend that the root canals must remain moist to benefit from the hydrophilic properties of sealers. However, they do not provide precise clinical instructions on how to achieve an ideal level of residual moisture before application of their products (Ehsani et al., 2014). Since no clear protocol is available for achieving an ideal state of residual moisture in the root canal (Zmener et al., 2008), different chemical agents such as different concentrations of alcohol have been tested for dentin moisture removal (Engel et al., 2005; Nagas et al., 2012; Stevens et al., 2006). Evidence shows that over‐drying may remove water from dentinal tubules and impair the efficacy of hydrophilic sealers and their adhesion to root dentin (Dias et al., 2014; Singh et al., 2019). The hydrophilicity of sealers is not enough for the ideal displacement of water in completely wet root canals, and the entrapment of water droplets at the sealer‐dentin interface compromises strong bonding. For the application of resin‐based sealers, such a high level of moisture contamination can also decrease the degree of conversion of monomers, and result in incomplete resin polymerization and reduction of bond strength to dentin. The solubility of sealers can also affect their sealing and adhesive properties (Nagas et al., 2012). However, there is no published data regarding the solubility of SureSeal Root and MTA‐Fillapex.

In the present study, although the air vacuum presented an interesting hydraulic drive, its dynamic was not effective as a drying system and yielded a lower PBS than ethanol and paper point, which was in line with the results of Paula et al. (2016).

The maximum mean PBS in the present study was recorded in SureSeal Root sealer followed by AH26, Endoseal MTA, and MTA‐Fillapex. The difference in PBS was significant among different sealers. Thus, the second null hypothesis of the study was also rejected.

Bioceramics are composed of spherical nanoparticles with maximum dimensions not exceeding 1.9 × 10^3^ µm. Considering their ability to penetrate into dentinal tubules and interact with dentin moisture, they are expected to have optimal dimensional stability and minimal shrinkage (Razmi et al., 2016). SureSeal Root is a calcium silicate‐based bioceramic sealer (Milani et al., 2021), which is supplied in premixed form (automix syringe), and is ready to use. Bioceramic sealers cause deposition of minerals, which results in formation of tag‐like structures within dentinal tubules (Reyes‐Carmona et al., 2009). calcium silicate‐based sealers can nucleate carbonated apatite since they can release calcium ions and maintain an alkaline environment. Therefore, the higher PBS shown by the bioceramic sealers may be due to higher amounts of released calcium ions, suggesting greater biomineralization at the dentin‐cement interface. The precipitation of apatite is proportional to the concentration of Ca^2+^ ions available (Retana‐Lobo et al., 2021).

On the other hand, calcium silicate‐based sealers have high flowability due to having small particles (<2 µm) and excellent viscosity, which enhance their penetration into dentinal tubules, anatomical irregularities, and gutta‐percha voids. Also, the calcium silicate content of SureSeal Root is responsible for its low or no shrinkage during its setting reactions (Nagas et al., 2012), which can result in its higher bond strength. The mean PBS of SureSeal Root was similar to that of AH26 and significantly higher than that of MTA‐Fillapex and Endoseal MTA. Due to their flowability, long polymerization time, and high affinity between the molecules (Khatib et al., 2019), epoxy resin‐based sealers can penetrate deeper into dentin irregularities (Demiriz et al., 2016; Eldeniz et al., 2005; Khatib et al., 2019; Lee et al., 2002), and have higher displacement resistance (Milani et al., 2021). Such inherent physicochemical properties may explain higher PBS and better adaptation at the adhesive interface in the AH26 group. AH26 has high creeping potential, which enhances its penetration into tiny irregularities, and bonding of sealer to dentin, due to the cohesion between the molecules. Accordingly, it increases the adhesion and resistance against sealer displacement on the dentin surface (Kurup et al., 2021). A recent meta‐analysis by Chopra et al. (2021) found no significant difference in the bond strength of resin sealers with calcium silicate‐based sealers, which was in line with the present findings.

In the present study, the mean PBS of AH26 was similar to that of Endoseal MTA and significantly higher than that of MTA‐Fillapex, which was consistent with the results of Kurup et al. (2021) and Paula et al. (2016). MTA‐Fillapex is supplied in the form of two pastes (Kurup et al., 2021). Controversy exists regarding its bond strength to root dentin. Sagsen et al. (2011) demonstrated that MTA‐Fillapex yielded the lowest PBS to root dentin, compared with epoxy resin and calcium silicate‐based sealers. Nagas et al. (2012) reported that MTA‐Fillapex had a lower PBS to root dentin than iRoot SP. Their results were in agreement with the present findings.

Regarding the mode of failure, the present results revealed that adhesive and mixed failures were the most common in all groups. Adhesive failure at the sealer‐dentin interface clearly underlines inadequate bond strength of sealer to dentin. The same results were reported by Nagas et al. (2012). In the present study, adhesive and mixed failures were the most common in AH26 and MTA‐Fillapex, and cohesive and mixed failures were the most common in Endoseal MTA and SureSeal Root sealers. In a study by Singh et al. (2019) the majority of failures were cohesive and mixed in AH Plus, and adhesive and cohesive in EndoSequence BC and MTA‐Fillapex.

In total, the technique of drying based on the type of sealer should be carefully selected as a critical step in endodontic treatment (Ehsani et al., 2014). It appears that drying the canal with ethanol or paper point along with the use of bioceramic sealers can enhance the adhesion of sealer to dentin. However, since ethanol showed a superior performance to other methods only with AH26 and did not present a significant difference with paper points, the authors would not recommend this method in general, but only for AH26, because ethanol causes over‐drying of the canal and dentinal tubules, and subsequently impairs the efficacy of hydrophilic sealers and their adhesion to root canal walls (Singh et al., 2019). The reason for higher efficacy of ethanol with resin‐based sealers than with bioceramic sealers is that elimination of moisture leads to a higher success rate in resin‐based sealers because resin sealers are naturally hydrophobic, and moisture degrades their structure; whereas, the effect of moisture on bioceramic sealers is much lower due to their inherently hydrophilic nature (Nagas et al., 2012).

This study had an in vitro design. Thus, generalization of results to the clinical setting should be done with caution. Future in vivo studies on multi‐rooted teeth are recommended. Also, the role of the obturation technique in this regard should also be assessed in future studies.

## 5 CONCLUSION

In the present study, drying the canals with ethanol and paper points enhanced the PBS of sealers to root dentin. Also, irrespective of the drying technique, SureSeal Root yielded the highest PBS to root dentin. In total, in dried canals, SureSeal Root showed the best results, while in wet canals, no difference existed among the sealers and they all had an equal performance.

## AUTHOR CONTRIBUTIONS

*Study concept and design*: Ahmadreza Sarrafan. *Acquisition of data*: Ahmadreza Sarrafan. *Analysis and interpretation of data*: Ahmadreza Sarrafan. *Drafting of the manuscript*: Ahmadreza Sarrafan. *Critical revision of the manuscript for important intellectual content*: Ali Soleymani. *Statistical analysis*: Seyedali Seyedmajidi. *Administrative, technical, and material support*: Tasnim Bagheri Chenari. *Study supervision*: Ali Soleymani.

## CONFLICT OF INTEREST STATEMENT

The authors declare no conflict of interest.

## Data Availability

The data used to support the findings of this study were supplied by the corresponding author under license and data will be available on request. Requests for access to these data should be made to the corresponding author.
